# Long-term *in vivo* polychlorinated biphenyl 126 exposure induces oxidative stress and alters proteomic profile on islets of Langerhans

**DOI:** 10.1038/srep27882

**Published:** 2016-06-13

**Authors:** Rodrigo Azevedo Loiola, Fabyana Maria dos Anjos, Ana Lúcia Shimada, Wesley Soares Cruz, Carine Cristiane Drewes, Stephen Fernandes Rodrigues, Karina Helena Morais Cardozo, Valdemir Melechco Carvalho, Ernani Pinto, Sandra Helena Farsky

**Affiliations:** 1 Department of Clinical and Toxicological Analyses, Faculty of Pharmaceutical Sciences, University of São Paulo, São Paulo, Brazil; 2 Fleury Group, São Paulo, Brazil

## Abstract

It has been recently proposed that exposure to polychlorinated biphenyls (PCBs) is a risk factor to type 2 diabetes mellitus (DM2). We investigated this hypothesis using long-term *in vivo* PCB126 exposure to rats addressing metabolic, cellular and proteomic parameters. Male Wistar rats were exposed to PCB126 (0.1, 1 or 10 μg/kg of body weight/day; for 15 days) or vehicle by intranasal instillation. Systemic alterations were quantified by body weight, insulin and glucose tolerance, and blood biochemical profile. Pancreatic toxicity was measured by inflammatory parameters, cell viability and cycle, free radical generation, and proteomic profile on islets of Langerhans. *In vivo* PCB126 exposure enhanced the body weight gain, impaired insulin sensitivity, reduced adipose tissue deposit, and elevated serum triglycerides, cholesterol, and insulin levels. Inflammatory parameters in the pancreas and cell morphology, viability and cycle were not altered in islets of Langerhans. Nevertheless, *in vivo* PCB126 exposure increased free radical generation and modified the expression of proteins related to oxidative stress on islets of Langerhans, which are indicative of early β-cell failure. Data herein obtained show that long-term *in vivo* PCB126 exposure through intranasal route induced alterations on islets of Langerhans related to early end points of DM2.

Over the last three decades, incidence of type 2 diabetes mellitus (DM2) has reached epidemic proportions worldwide^1^. Although excessive caloric consumption and sedentary lifestyle are well recognized risk factors for DM2, recent evidence has suggested that exposures to persistent organic pollutants (POPs) may contribute to the rising risk of metabolic disorders^2^,^3^. POPs make up a broad class of chemical organic lipophilic compounds of natural or anthropogenic origins that are resistant to the environmental degradation, including aldrin, dieldrin, dioxins, furans, and polychlorinated biphenyls (PCBs)^4^.

PCBs cover a class of 209 congeners widely employed in industrial processes until the late 1980 s, when their production was banned. By then, large quantities of PCBs had been released into the environment worldwide, and high concentrations of PCBs have been detected in the water, air, food, animals, and even human milk^5^,^6^. According to Toxic Equivalency Factor (TEF), the PCB126 (3,3′,4,4′,5-pentachlorobiphenyl) is considered the most toxic PCB congener, once its biological effects are comparable to those evoked by 2,3,7,8-tetrachlorodibenzo-p-dioxin (TCDD; TEF = 0.1)^7^. In this regard, studies have clearly showed that chronic PCB126 exposure causes several toxic effects, including cancer development and impairment of liver, lung, and cardiovascular functions^7^. PCB126, a co-planar or dioxin-like PCB agent, activates genomic pathways by binding and activating the cytoplasmatic aryl hydrocarbon receptor (AhR). The consequent agonist-AhR complex translocates into the nucleus and heterodimers with the AhR nuclear translocator (ARNT). The complex AhR:ARNT activates the promoters of genes containing xenobiotic response elements (XRE) that induce the transcription and expression of AhR target genes^8^.

Although diet continues to be an important source of PCB intoxication, studies have shown that inhalation is a relevant route of PCB exposure. Because of their physical-chemical characteristics, PCBs are dispersed in the air and soil and are also sorbed to particulate matter^9^. Moreover, the use of building materials containing PCBs has contributed to high levels of indoor air contamination. Indeed, the positive correlation of longer time in contaminated indoor areas and endogenous accumulation of PCB has been shown^10^,^11^,^12^. Despite these data, the inhalation route remains a neglected pathway of exposure, and studies addressing the health outcomes of inhaled PCBs exposure must be considered in risk assessment^11^. Recently, our group demonstrated that long-term *in vivo* PCB126 exposure by intranasal instillation into Wistar rats induced the expression of AhR in in the liver, lungs, kidneys, and adipose tissue, and PCB126 levels were found in the lungs and liver. Moreover, intranasal PCB 126 exposure affected the G protein coupled receptor (GPCR) signaling in circulating leukocytes of rats and consequently impaired innate immunological functions related to the host’s defense to infections^13^.

The role of PCB126 on metabolic disease is not fully described, but it nevertheless has been shown that *in vivo* PCB126 exposure induces alterations in lipid^14^,^15^ and glucose metabolism^16^ and evokes chronic inflammation and atrophy of exocrine pancreas^17^. However, to our knowledge, the toxicity of the inhalatory pathway of PCB126 exposure on endocrine pancreas, a pivotal tissue to regulation of glucose and lipid homeostasis, have not been investigated. Therefore, we characterized the effects of long-term *in vivo* PCB126 exposure via intranasal instillation on metabolic and cellular parameters of rats and investigated the toxic mechanism on islets of Langerhans. Data obtained showed evident metabolic alterations caused by PCB126 exposure, and that oxidative stress and alteration on protein profile expression on islets of Langerhans are early end points of PCB126 intoxication.

## Results

### *In vivo* PCB126 exposure induced body weight gain independently on accumulation of periepididymal and retroperitoneal adipose tissues

**Figure 1:**
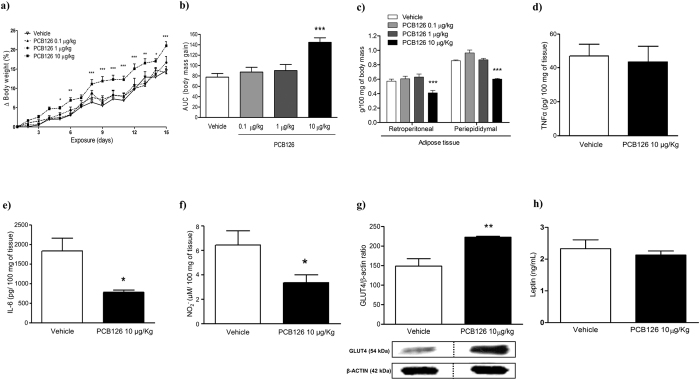
Effects of *in vivo* PCB126 exposure on body weight and lipid metabolism. (**a**) Lines graph showing weight gain (% in relationship to the beginning of exposure) and (**b**) bars graph showing AUC of body weight gain during PCB126 exposure (0.1, 1 or 10 μg/kg). (**c**) Mass (g/100 g of body weight) of retroperitoneal and periepidymal adipose tissue. Bars graphs showing levels of TNF-α (**d**), IL-6 (**e**), and NO (**f**) on retroperitoneal adipose tissue homogenates. (**g**) Bars graph and representative image of GLUT4 expression on adipose tissue. Data were analyzed by two-way ANOVA (**a**); one-way ANOVA (**b,c**); or student’s *t*-test (**d–h**). *P < 0.05; **P < 0.01; ***P < 0.001 vs. vehicle group.

Vehicle or PCB126 exposed rats gained body weight from the 1^st^ until the 15^th^ day of exposures (Fig. 1a); nevertheless, the body weight gain was significantly higher in rats exposed to PCB126 (10 μg/kg) compared to rats exposed to vehicle or to lower doses of PCB126 (0.1 or 1 μg/kg; Fig. 1a,b). The gain of weight in PCB126 exposed rats was not associated to alterations in food intake and water consumption, once these parameters were similar between all experimental groups (Fig. S1). It is important to mention that the gain of weight was not detected in rats exposed to PCB180, a non-dioxin PCB, following the same protocol of exposure to PCB126 (Fig. S2). The exacerbated gain of weight detected in PCB126 exposed rats was also not dependent on adipose tissue accumulation, because PCB126 exposure evoked a significant reduction in the mass of periepididymal and retroperitoneal adipose tissue (Fig. 1c). Moreover, PCB126 exposure did not cause inflammation in the retroperitoneal adipose tissue, as it had no effect in the amount of tumor necrosis factor-α (TNF-α; Fig. 1d), and reduced the levels of interleukin-6 (IL-6; Fig. 1e) and nitrite (Fig. 1f), the main metabolite of nitric oxide (NO). On the other hand, the expression of GLUT4, the insulin-regulated glucose transporter, was markedly enhanced in retroperitoneal adipose tissue from rats exposed to the higher dose of PCB126 (Fig. 1g). Leptin levels on adipose tissues were not altered (Fig. 1h).

### *In vivo* PCB126 exposure caused toxicity in the liver and kidney and altered lipid metabolism

Although it has been shown that PCB126 causes liver toxicity by enhancing cytochrome P450 and decreasing glutathione levels via AhR binding^18^,^19^, our data show for the first time that *in vivo* PCB126 inhalatory exposure elevated levels of serum creatinine and gamma-glutamyl transpeptidase (GGT), and total protein in urine (Table 1).

Nevertheless, levels of aspartate aminotransferase and alanine aminotransferase were not altered by PCB126 intoxication (Table 1). Therefore, we infer that the schedule of *in vivo* PCB126 exposure was not intense enough to cause liver damage and we foresee that longer exposure and/or higher dose of PCB126 must be employed to induce severe hepatic injuries. Moreover, *in vivo* PCB126 exposure also induced a significant elevation of serum levels of triglycerides, cholesterol, and very low density lipoprotein (VLDL) compared to animals exposed to vehicle (Table 1).

### *In vivo* PCB126 exposure altered glucose metabolism

**Figure 2:**
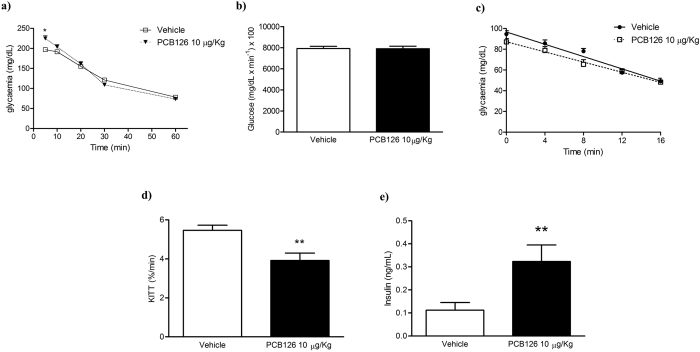
Effects of *in vivo* PCB126 10 μg/kg exposure on metabolic parameters. (**a**) Glycaemia measured during GTT and (**b**) determination of AUC; (**c**) glycaemia measured during ITT and (**d**) KITT. Serum levels (ng/mL) of insulin (**e**). Data were analyzed by student’s *t*-test. **P < 0.01; ***P < 0.001 vs. vehicle group.

Fasting glycaemia in rats exposed to PCB126 was compared to that in vehicle exposure animals (Table 1). Furthermore, the AUC of glucose tolerance test (GTT) was similar between the experimental groups (Fig. 2b), although we observed higher glycemic peak in rats exposed to PCB126 compared to vehicle five minutes after IV administration of glucose (Fig. 2a). In addition, *in vivo* PCB126 exposure significantly reduced insulin sensibility (Fig. 2c,d) and increased serum levels of insulin (Fig. 2e) compared with rats exposed to vehicle. It is noteworthy to mention that levels of insulin in naïve rats is similar to those observed in vehicle treated rats (0.15–0.18 ng/mL).

### *In vivo* PCB126 exposure did not alter islet of Langerhans morphology and viability, but exacerbated free radical production

**Figure 3:**
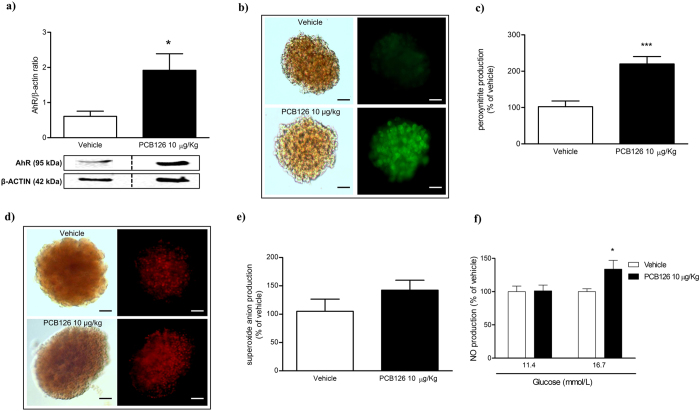
Effects of *in vivo* PCB126 10 μg/kg exposure on free radical generation on islets of Langerhans. (**a**) Bars graph and representative image of AhR expression on islets of Langerhans. Representative image and bars graph showing peroxynitrite (**b,c**) and superoxide anion production (**d,e**). Determination of NO production (**f**) in islets cultured at physiological (11.4 mM) or high glucose levels (16.7 mM). Data were analyzed by student’s *t*-test (**a,c,e**) or two-way ANOVA (**f**). *P < 0.05; ***P < 0.001 vs. vehicle group.

*In vivo* PCB126 exposure evoked significant increase in protein expression of AhR on islets of Langerhans (Fig. 3a), but had no effect on insulin secretion (Fig. S4A), islets morphology (Fig. S4B), viability (Fig. S4C), or cell cycle (Fig. S4D). Nevertheless, *in vivo* PCB126 exposure evoked a significant exacerbation of peroxynitrite generation on islets of Langerhans (Fig. 3b,c) while having no effect on superoxide anion production (Fig. 3d,e). *Ex vivo* incubation of islets of Langerhans with different concentrations of glucose showed that at physiological glucose levels (11.4 mM), the NO production was similar in islets collected from vehicle or PCB126 exposed rats; nevertheless, significant increases in NO levels in islets from rats exposed to PCB126 were detected when islets were cultured in high glucose medium (16.7 mM) (Fig. 3f).

### *In vivo* PCB126 exposure did not alter inflammatory parameters in pancreas

**Figure 4:**
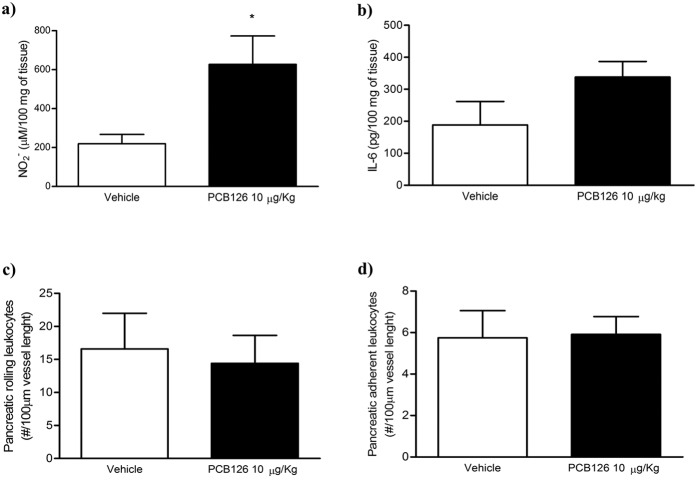
Effects of *in vivo* PCB126 10 μg/kg exposure on pancreatic inflammatory parameters. Determination of NO (**a**) and IL-6 (**b**) levels on pancreatic homogenates. Determination of rolling (**c**) and adherent (**d**) leukocytes in pancreatic microcirculation. Data were analyzed by student’s *t*-test. *P < 0.05 vs. vehicle group.

Only elevated NO levels were observed in pancreatic homogenates of rats exposed to PCB126 (Fig. 4a). IL-6 levels (Fig. 4b) and numbers of rolling (Fig. 4c) or adherent leukocytes (Fig. 4d) on pancreatic microcirculation were not altered by PCB126 exposure. Moreover, PCB126 exposure did not alter plasma cytokines interleukin 1 β (IL-1β), IL-6, or tumor necrosis factor (TNF-α) (Fig. S3).

### *In vivo* PCB126 exposure altered proteomic profile on islets of Langerhans

**Figure 5:**
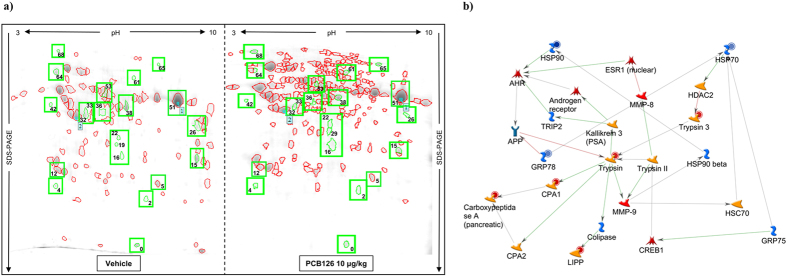
Effects of *in vivo* PCB126 10 μg/kg exposure on proteomic profile of islets of Langerhans. (**a**) Representative image of 2D gel stained with Coomassie Blue R 350 loaded with protein from islets isolated from rats exposed to vehicle or PCB126 10 μg/kg. Matched spots that showed significant changes in protein expression levels were highlighted. (**b**) Figure generated by software Metacore^®^ showing relationship between proteins identified by proteomics analysis. Red circle: down-regulated by PCB126 exposure; blue circle: up-regulated by PCB126 exposure. LIPP: pancreatic triacylglycerol lipase; CPA: carboxypeptidase A1; HSP: heat shock protein; AhR: aryl-hydrocarbon receptor.

Fresh islets of Langerhans samples from experimental groups were submitted to 2D electrophoresis (Fig. 5a). We identified 105 and 201 spots in the vehicle and PCB126 exposed group, respectively. After a quantitative analysis, a 1.4-fold change in protein expression was adopted as a cut-off to consider changes between groups statistically significant. We found that 22 pairings satisfied this condition. Matched spots are represented in Table 2. Overall, it is interesting to note that *in vivo* PCB126 exposure up-regulated 8 and down-regulated 14 proteins. To better understand the way in which they are engaged against the influence of the PCB126, the biochemical pathways of these proteins were evaluated using the software suite for functional analysis MetaCore^®^ (Fig. 5b). Altered proteins on islets from rats exposed to PCB126 possibly are related to oxidative stress and failure of β-cells, namely, Heat Shock protein 90 kDa (HSP90), Electron transfer flavoprotein subunit alpha (Alpha-ETF), 78 kDa glucose-regulated protein (GRP78), nucleoside diphosphate kinase A and B (NDKA/NDKB), and ribonuclease inhibitor (Table 2 and Fig. 5b).

## Discussion

Although the current literature has suggested that exposure to PCBs may contribute to the genesis of DM2^2^,^3^,^20^, the mechanisms responsible for the toxic effect have not been fully understood. Therefore, by using an intranasal long-term PCB126 exposure into rats, we showed systemic toxicity, by altering lipid and glucose metabolisms and oxidative stress on islets of Langerhans, the endocrine portion of pancreas. Moreover, PCB126 exposure modified the expression of proteins on islets of Langerhans, leading to a profile of proteins closely correlated to failure of pancreatic β-cells.

Obesity is one factor responsible for the genesis of DM2, and a positive correlation of POP exposures and obesity has been fully described in the contemporary literature^21^,^22^. Indeed, we here confirmed that PCB126 exposure induces gain of weight, but it was not associated with the enhanced retroperitoneal or periepididymal fat depots or to enhanced food and water intake. Indeed, levels of leptin in adipose tissue were not altered. Conversely, similar exposure to non-co-plannar PCB 180 did not lead to weight gain, which would be expected considering that PCB180 exposure is associated with obesity in humans^23^. It is noteworthy that both PCBs 126 or 180 were well absorbed by animals in our studies, because higher levels of PCB126 or PCB180 were found in different tissues of exposed animals, and PCB126 exposure induced AhR expression in different tissues, as liver, adipose tissue, kidney, and lungs^13^.

The gain of weight after PCB126 exposure may be due to hydric retention, as creatinine levels in the plasma and total proteins in urine were elevated after the PCB126 exposure. The *in vivo* renal toxicity caused by PCB126 is shown here for the first time, as only *in vitro* studies had already demonstrated the ability of PCBs, especially non-coplannar congeners, to induce apoptosis in cell kidney cultures^24^.

The physiological role of AhR in regulating body weight and insulin sensitivity, and mediating environmental toxicant-induced obesity, insulin resistance, and development of diabetes has been fully shown^25^,^26^. Nevertheless, the molecular mechanisms underlying the AhR activation on metabolic diseases are very complex, and the outcomes vary during the development of diseases. In this context, we previously showed that *in vivo* inhalatory PCB126 exposure enhanced AhR expression in the adipose tissues^13^, and here we suppose that both reductions on periepididimal and retroperitoneal fat depots detected in PCB126 exposed rats may be due to direct activation of AhR in the adipocytes. This hypothesis is based on recent data showing that AhR null mice exhibited increased fat mass when fed a standard, low or high fat diet^27^; transgenic mice with constitutively activated AhR present decreased adipose tissue deposits associated with elevation of peripheral fat mobilization^28^; and *in vitro* ligand-activated AhR inhibits the lipid synthesis, adipocyte differentiation, and secretion of adypocyte proliferating mediators^14^,^29^,^30^,^31^. On the other hand, AhR activation induces inflammation in adipose tissue, contributing to obesity^27^,^28^,^32^,^33^,^34^. PCB126 exposure by intranasal route did not induce secretion of inflammatory cytokines in the adipose tissue and plasma, excluding an apparent inflammatory state as a hallmark of PCB126 intoxication by intranasal route. Together, our data suggest that weight gain is not due pro-adipogenic actions of PCB126 and may reflect hydric retention by kidney toxicity. We suppose that longer exposure, higher dose or different via of administration may be required to evoke the gain of adipose tissue after PCB126 exposure, as mentioned previously in literature^17^.

Meanwhile, biochemical analysis of serum clearly showed the effects of PCB126 exposure on lipid and glucose metabolism, reinforcing the interference of PCBs on both gluconeogenesis and fatty acid oxidation in the liver, which precedes PCBs hepatic toxicity^14^,^15^,^35^,^36^. In this context, it is important to mention that elevated levels of serum GGT, as here induced by PCB126 exposure, have been also considered an early biomarker for development of DM2^37^,^38^,^39^. Furthermore, PCB126 exposure up-regulated the expression of GLUT-4 in the retroperitoneal adipose tissue, which was accompanied by marked reduction of insulin sensibility and elevated levels of serum insulin. Taken together, data obtained show that *in vivo* PCB126 exposure induces insulin resistance and pre-diabetic state, and we infer that GLUT-4 up-regulation may be a compensatory effect in response to insulin resistance.

The pathophysiological hallmarks of DM2 consist of insulin resistance and β-cell dysfunction^40^. At early stages, β-cells compensate for insulin resistance by up-regulating the secretion of the hormone to maintain physiologic glycemic levels^41^. The *in vivo* PCB126 exposure enhanced serum insulin levels, suggesting that normal glucose tolerance in our experimental model results from up-regulation of insulin secretion by β-cells. Moreover, *in vivo* PCB126 exposure up-regulated AhR expression on isolated islets of Langerhans, showing that endocrine pancreas is also a target of PCB126 intoxication. To our knowledge, the up-expression of AhR by POPs on islets of Langerhans is shown here for the first time. Long-term stimulation of islets of Langerhans can lead to β-cell failure, which may result from inadequate expansion of mass or failure of existing β-cells to respond to glucose^41^. Our data excluded the PCB126 actions on proliferation and viability of islets of Langerhans; nevertheless, PCB126 exposure enhanced peroxynitrite production by islets, an end-point of oxidative stress. Although both superoxide and NO levels were similar between the groups at basal conditions, *in vivo* PCB126 exposure exacerbated NO production in islets cultured in high glucose medium. Indeed, the NO levels were also elevated on pancreatic homogenates from rats exposed to PCB126. This may be an important mechanism of PCB126 actions, since NO inhibits insulin secretion induced by glucose^42^ and chronic exposure to high levels of NO promotes β-cell death^43^.

Increasing evidence has suggested that a subclinical inflammation is involved in impairment of β-cell function in the course of DM2^44^,^45^. Indeed, it has previously been reported that *in vivo* PCB126 exposure induces chronic inflammation on exocrine pancreas^17^. Conversely, we did not find any signal of inflammation in the pancreas because the interaction of leukocytes into endothelial cells of pancreatic microcirculation and the production of cytokines were not modified by PCB126 exposure. It is noteworthy that leukocyte interaction into pancreas microcirculation is a hallmark to initial phases of pancreas inflammation. Intravital microscopy is a useful technique to identify the *in viv*o role of this phenomenon to evolution of the inflammatory response^46^,^47^.

Proteomic analysis has largely contributed to the knowledge of the phenotype of cells in different conditions^48^, and we here employed this experimental tool to better detect earlier end-point of *in vivo* PCB126 exposure. Data obtained highlighted 22 different proteins between the experimental groups, 8 of which were up-regulated and 14 of which were down-regulated by PCB126 exposure. Among the up-regulated proteins altered by PCB126 exposure, HSP90, Alpha-ETF, and GRP78 are related to oxidative stress. In this regard, a positive association was previously reported between DM2 and the up-regulation of HSP90^49^ on islets of Langerhans, since the overexpression of HSP90 on islets has been associated with elevation of β-cell ATP-sensitive potassium (K_ATP_) channel expression^50^, enhanced insulin secretion, and promotion of glucokinase activation, the rate-limiting enzyme in glycolysis^51^. The up-regulation of Alpha-ETF also seems to be beneficial in response to PCB126 exposure, since it was previously reported that its down-regulation reduces respiratory chain in islets from an animal model of DM2, leading to mitochondrial dysfunction and oxidative stress^52^. On the other hand, the up-regulation of GRP78 probably represents a deleterious effect of PCB126 exposure. Previous studies have reported a positive association between up-regulation of GRP78 and reduction of insulin secretion, reticulum endoplasmic stress^53^, and development of DM2^54^. In the same way, the down-regulation of both NDKA/B and ribonuclease inhibitor possibly represents an early sign of β-cell damage in response to PCB126 exposure. In this respect, previous studies reported the down-regulation of NDKA/B in islets of Langerhans from a rat model of DM2^55^ and in insulin-secreting β-cells exposed to high glucose conditions^56^, suggesting that NDKA/B expression is reduced in islets submitted to glucolipotoxic conditions. Similarly, the down-regulation of ribonuclease inhibitor probably favors the exacerbation of free radical production on islets, since it has been demonstrated that this protein plays a key role as a defensive system against oxidative stress^57^. Taken together, the proteomic analysis suggests that up-regulation of HSP90 and Alpha-ETF possibly occurred to compensate for the insulin resistance induced by PCB126 exposure and played a protective role against oxidative stress. On the other hand, the up-regulation of GRP78 and the down-regulation of NDKA/B and ribonuclease inhibitor can represent early signs of β-cell failure induced by PCB126 exposure.

In conclusion, our study presents the first findings indicating that long-term *in vivo* PCB126 exposure through intranasal route induces toxic systemic effects associated with early end-points of DM2, including insulin resistance, hypertriglyceridemia, hyperinsulinemia, and oxidative stress on islets of Langerhans. A proteomic approach revealed that *in vivo* PCB126 exposure regulated the expression of some proteins (HSP90 and Alpha-ETF) to compensate for the insulin resistance and protect against oxidative stress, while others (GRP78, NDKA/B and ribonuclease inhibitor) may represent an early sign of β-cell failure. Taken together, our study highlights the influence of environmental pollutants on progression/development of DM2 and can help guide the adoption of new health public policies.

## Material and Methods

### Chemicals

All reagents were purchased from Sigma-Aldrich (St. Louis, MO, USA) unless otherwise stated. PCB126 (purity 99%) was purchased from AccuStandard (New Haven, CT, USA) and antibodies from Abcam (Cambridge, MA, USA). Reagents for cell culture were obtained from Vitrocell (Campinas, SP, Brazil), and ketamine and xylazine were purchased from Vetbrands (Jacarei, SP, Brazil). Annexin-V FITC-conjugated antibody, IL-1β, IL-6, and TNF-α ELISA kits were purchased from BD Pharmingen (San Diego, CA, USA). Only ELISA insulin kit was purchased from Millipore (Billerica, MA, USA). Chemiluminescence detection solution was purchased from BioRad (CA, USA). BCA kit was obtained from Pierce^®^ (Walthan, MA, USA).

### Animals

Male Wistar rats (180-220 g) were purchased from the animal facility of Chemistry Institute at the University of São Paulo. The animals were fed a standard pellet diet and water, *ad libitum*. All procedures were performed according to the Brazilian Society for the Science of Laboratory Animals (SBCAL) and approved by the Institutional Animal Care and Use Committee from Faculty of Pharmaceutical Sciences – University of Sao Paulo (Protocol number 315).

### Protocol of *in vivo* PCB126 exposure

For 15 days, once a day, the animals were anaesthetized with ketamine/xylazine solution (80:8 mg/kg respectively, intraperitoneally, i.p.) and exposed to PCB126 0.1; 1 or 10 μg/kg of body weight by intranasal instillation, corresponding to 0.0001, 0.001, and 0.01 ppm, respectively. Control animals received the equivalent volume of vehicle (saline solution containing 0.5% of dimethyl sulfoxide — DMSO). During exposure, the body weight, food intake, and water consumption were daily measured. Five hours after the last vehicle or PCB126 exposures, animals were anesthetized with pentobarbital sodium (40 mg/kg; i.p.) instead ketamine/xylazine, since previous studies have shown that pentobarbital has less pronounced effects on basal glycaemia and other metabolic parameters^58^. Then, animals were euthanized and samples (blood, adipose tissue, pancreas, and liver) were collected.

### Biochemical quantification

Basal glycaemia in conscious animals was detected in a sample of blood collected from the tail vein using a glucometer (Accu-check Active^®^, Roche, Switzerland). Blood samples to determine other biochemical parameters were collected from abdominal aortae of anaesthetized animals (pentobarbital sodium, i.p.), and the serum was isolated by centrifugation (1500 *g*, for 10 min, at 4 °C). Insulin was quantified by ELISA, while cholesterol, triglycerides, alanine aminotransferase (ALT), aspartate aminotransferase (AST), gamma-glutamyl transpeptidase (GGT), urea, and creatinine were quantified by enzymatic method in automated equipment (Labmax240^®^, Labtest, Brazil).

### Glucose and insulin tolerance test

For both insulin tolerance test (ITT) and glucose tolerance test (GTT), glycaemia was measured with a blood sample obtained from the tail. For ITT, insulin (0.75 U/kg) was intravenously (IV) administered and glycaemia was measured over time (0, 4, 8, 12, and 16 min), as previously described^59^. The fall of glucose levels in the first 15 minutes after i.v. insulin administration is a function of insulin-stimulated glucose uptake by tissues and its ability to suppress glucose output by the liver (shorter ITT measurement). For this reason, we carried out a shorter version of ITT, since the systemic counter-regulatory hormone response to hypoglycemia, which can interfere with insulin sensitivity, occurs only after 20 minutes of the insulin injection (longer ITT measurement)^60^. The coefficient of glucose disappearance rate (KITT) was calculated as the slope of linear regression of glucose concentrations determined after insulin administration. GTT was administered as 375 mg/mL of glucose (0.2 mL/100 g of body weight) through IV injection, and glycaemia was measured over time (0, 5, 10, 20, 30, and 60 min). The index of glycaemia decay (mg/dL × min^−1^) × 100 was calculated as the area under the curve (AUC).

### Isolation of islets of Langerhans

After anesthesia (pentobarbital sodium; 40 mg/kg; i.p.), blood was collected, followed by euthanasia (exsanguination). The common bile duct was cannulated, and the pancreas was inflated with digestion solution Hanks balanced salt solution (HBSS) containing 0.7 mg/mL type V collagenase. Then, the pancreas was excised, picked, transferred to a Falcon^®^ tube (50 mL) containing 10 mL of digestion solution, and incubated at 37 °C for 25 min. After incubation, samples were centrifuged (800 rpm, 3 min), followed by purification of islets with Histopaque^®^-1077 gradient. Subsequently, islets were examined using a dissecting microscope and were hand-picked.

### Free radicals generation on islets of Langerhans

Freshly isolated islets were incubated (30 min at 37^o^ C) with HBSS containing fluorescent probes to detect either peroxynitrite (2,7-dichlorodihydrofluorescein diacetate—DCFH-DA, 30 μM) or superoxide anion (dihydroethidium—DHE, 10 μM). After incubation, images of islets were obtained using a fluorescent microscope (Axiovert 100 M, Carl Zeiss, Germany). Fluorescence intensity (optical density) was determined by using the software AxioVision Release 4.8 and expressed as arbitrary units (a.u.). For nitric oxide (NO) measurement, islets (40 islets/rat) were cultured (24 hr at 37^o^ C, 5% CO_2_) in RPMI medium containing 10% FBS and physiological (11.1 mM) or high (16.5 mM) levels of glucose. The NO_2_^−^ levels were determined by performing the Griess reaction assay. The values of peroxynitrite, superoxide anion, and NO production by islets obtained from PCB126 exposed rats were expressed as percentage (%) in relationship to islets isolated from vehicle exposed rats (100%).

### Western Blot assay

The western blot for detection of AhR and GLUT-4 on islets of Langerhans or adipose tissue, respectively, was carried out as previously reported^13^. Briefly, samples were suspended in RIPPA buffer containing inhibitor protease cocktail, sonicated, and centrifuged (12.000 rpm at 4 °C for 20 min). Total protein content was determined by Bradford’s method, and samples were mixed to Laemmli’s sample buffer. Samples (50 μg) were loaded in SDS-PAGE (10%), submitted to electrophoresis, and transferred to a nitrocellulose membrane in a transfer apparatus (Bio-Rad Laboratories, CA, USA). Membranes were incubated in blocking buffer (TBS-T — 20 mM Tris; 137 mM NaCl; 0.02% Tween 20 — containing 5% nonfat dry milk) followed by incubation (overnight at 4 °C) with monoclonal antibodies anti-AhR (1:1000) or GLUT-4 (1:500). Sequentially, membranes were incubated with peroxidase-conjugated secondary antibody (1:10000). Membranes were incubated with chemiluminescence detection solution, and immunoreactive bands were detected by autoradiography exposition. Band intensities were determined by optical densitometry using the software Image J (NIH, USA). The expression of actin was used as loading control.

### Assessment of inflammatory parameters

For determination of leukocyte-endothelium interaction on pancreatic microcirculation, animals were anesthetized with ketamine/xylazine solution (80:8 mg/kg respectively, intramuscular injection - IM), and a portion of the pancreas was surgically exteriorized to allow observation of the microcirculation. Animals remained on a special board thermostatically controlled at 37 °C, and the preparation was washed with Ringer-Locke solution (pH 7.4 at 37 °C) containing 1% gelatin. Images of second-order venules (14–18 μm of diameter) were obtained using an intravital microscopy system (Axioplan II, Carl-Zeiss, Germany) and captured with a video camera (AxioCam HRc, Carl-Zeiss). The number of adherent leukocytes (those that kept still for at least 30 sec) per 100 μm venule length and of the rolling leukocytes was monitored under basal conditions for 10 min.

To determine inflammatory mediators, pancreas and retroperitoneal adipose tissue were homogenized and suspended in RIPPA buffer containing protease inhibitors. NO levels were determined by Griess reaction, while IL-6 and TNF-α levels were determined by ELISA commercial kit.

### Proteomic data of pancreatic islets

#### Digestion and protein determination

Isolated islets (2500, pool of 10 animals) were suspended in RIPPA buffer, homogenized, and centrifuged (16000 *g*, 30 min, 4 °C). The supernatant was collected and concentrated in SpeedVac (Concentrator 5301, Eppendorf^®^, Hamburg, Germany), resuspended in PBS containing protease inhibitor cocktail. The protein determination was performed using the BCA kit.

#### 2D Electrophoresis

##### Sample Preparation

Proteins (350 μg) were precipitated with 1 volume of 20% (weight/volume - wt/vol) trichloroacetic acid, 0.07% (volume/volume - vol/vol) β-mercaptoethanol in acetone (−20 °C, 60 min), and washed 3 times with 0.07% (vol/vol) β-mercaptoethanol in acetone. Pellets were resuspended in rehydration buffer (2% Pharmalyte pH 3–10 in DeStreak Rehydration Solution, GE Healthcare).

##### Isoelectric Focusing (IEF)

Immobiline/IPG strips (pH 3–10, 7 cm—GE Healthcare) were rehydrated overnight in rehydration buffer containing protein samples (250 μg/strip). Strips were subjected to IEF on Ettan IPGphor 3 apparatus (GE Healthcare) under the following protocol: 300 V/200Vh; 300–1000 V/200 to 300Vh; 1000 V to 5000 V/300–4000 Vh e 5000 V/1250Vh.

##### SDS-PAGE

After IEF, strips were incubated in equilibration buffer Urea 6 M, Tris-HCl pH 8.8 75 mM, 30% (vol/vol) Glycerol, 2% (wt/vol) SDS containing 1.5% (wt/vol) DTT in the first step and 2.5% (wt/vol) iodoacetamide during the second step (15 min per step). The SDS-PAGE was carried out using 12.5% polyacrylamide gels (10 × 8 cm) on a SE 260 mini-vertical gel electrophoresis unit (Amersham Biosciences) under the following protocol: 80 V/30 min + 200 V/75 min. Then, gels were fixed with 50% (vol/vol) methanol, 10% (vol/vol) acetic acid solution, and stained with Coomassie Blue R 350 (GE Healthcare). Stained gels were scanned using an ImageScanner III scanner and LabScan software (GE Healthcare).

##### Gel Analysis

Images were analyzed using Image Master Platinum software (v.7.0, GE Healthcare). Spots were detected automatically without filtering, and gel patterns were matched between groups. At least a 1.4-fold change in the relative abundance between matched spots was considered significant, and these protein spots were submitted to trypsin digestion and analysis by liquid chromatography tandem mass spectrometry (LC-MS/MS).

#### Trypsin Digestion

Protein spots were washed (water and 50% acetonitrile) and then reduced (50 mM ammonium bicarbonate containing 10 mM DTT) for 30 min at 56 °C and then alkylated (55 mM iodoacetamide) for 30 min. Excess iodoacetamide was neutralized using 5 mM DTT. After washes with 50 mM ammonium bicarbonate, proteins were digested overnight with sequencing grade modified trypsin (enzyme/protein ratio 1:50) at 37 °C. The digest was evaporated to dryness in a SpeedVac and then kept at −80 °C until analysis.

#### Qualitative analysis of peptides by LC-MS/MS

Each sample was suspended in 0.05% trifluoroacetic acid containing 5% acetonitrile and incubated for 10 min at 37 °C under stirring (1400 rpm). The chromatographic analysis were performed by in a system composed by a nanoACQUITY UPLC (Waters) system coupled with a hybrid Quadrupole-Orbitrap mass spectrometer (Q-Exactive, Thermo Fisher Scientific) equipped with a nano-electrospray ionization source and operating at positive mode. Samples were loaded onto a PST C18 nanoACQUITY Trap column (180 μm × 20 mm) with flow rate set to 15 μL/min of 0.1% (vol/vol) trifluoroacetic acid during 3 minutes. Analytic separation of the peptides was performed using a nanoACQUITY UPLC HSS C18 Column (1.8 μm, 75 μm × 150 mm) and a two-step linear gradient was used starting from 2% (vol/vol) dimethyl sulfoxide (DMSO) and 0.1% (vol/vol) formic acid and increasing to 5% (vol/vol) DMSO, 25% (vol/vol) acetonitrile, and 0.1% (vol/vol) formic acid over 45 minutes; followed by a 30-minute gradient of 5% (vol/vol) DMSO, 40% (vol/vol) acetonitrile, and 0.1% (vol/vol) formic acid. For each cycle, one full MS scan (m/z 390–1,650) was acquired in the Orbitrap at a resolution of 70,000 at m/z 200 with an automatic gain control target of 3e6. Each full scan was followed by the quadrupole selection and isolation of the 12 most intense ions at a window m/z 4, which were dissociated through higher-energy collisional dissociation, using normalized collision energy of 26 (MS/MS). The product ions were analyzed by the Orbitrap at a resolution of 17,500 at m/z 200 with an automatic gain control target of 1e5. Ions with an unassigned, 1, and >8 charges were rejected, and dynamic exclusion was set to 30 sec. Data were analyzed by MaxQuant software (Max-Planck Institute of Biochemistry) and compared with a database *Rattus norvegicus* (rat)—UniProtKB— taxonomy 10116 (http://www.uniprot.org/taxonomy/10116). Proteins were considered identified when at least two peptides were identified, of which at least one was uniquely assigned to the respective sequence. The False Discovery Rate (FDR) was adjusted to 1%^61^. The Maximum Missed Cleavage (MMC) considered was 2, and the amino acid isoleucine was taken as leucine. Finally, among the list of generated proteins were classified those with lower Posterior Error Probability (PEP), highest sequencing coverage rates, and higher ion intensity. The identified proteins were analyzed using MetaCore™ software (Thomson Reuters) to assess the biochemical processes and metabolic pathways in which the most expressed proteins would be involved.

### Statistical analysis

Results represent mean ± SE (n = 5–6 per group). Comparison between three or more groups were analyzed by one- or two-way ANOVA as appropriate, with *post hoc* comparisons using the Tukey or Bonferroni test, respectively. Comparisons between two groups were analyzed by student’s *t*-test. In all cases, P < 0.05 was considered as statistically significant.

## Additional Information

**How to cite this article**: Loiola, R. A. *et al*. Long-term *in vivo* polychlorinated biphenyl 126 exposure induces oxidative stress and alters proteomic profile on islets of Langerhans. *Sci. Rep.* **6**, 27882; doi: 10.1038/srep27882 (2016).

## Supplementary Material

Supplementary Information

## Figures and Tables

**Table 1:** Effects of *in vivo* PCB126 intranasal exposure (10 μg/Kg) on biochemical parameters and markers of renal and hepatic function.

Biochemical parameter *concentration*	Vehicle	PCB126 10 μg/kg
Glycaemia *mg/dL*	94.60 ± 3.23	87.14 ± 3.21
Triglycerides *mg/dL*	46.33 ± 2.14	106.2 ± 8.43***
Cholesterol *mg/dL*	69.60 ± 1.94	88.27 ± 1.62***
High density lipoprotein (HDL) *mg/dL*	61.22 ± 1.99	69.33 ± 4.618
Very low density lipoprotein (VLDL) *mg/dL*	11.00 ± 1.18	21.23 ± 1.69***
Gamma-glutamyl transpeptidase (GTT) *U/L*	5.67 ± 2.14	17.13 ± 1.84**
Aspartate aminotransferase (AST) *U/L*	86.67 ± 6.75	96.28 ± 11.26
Alanine aminotransferase (ALT) *U/L*	43.30 ± 2.34	39.91 ± 6.32
Urea *mg/dL*	42.64 ± 2.26	49.35 ± 3.17
Creatinine *mg/dL*	0.6 ± 0.06	1.3 ± 0.08***
Total protein (urine) *g/dL*	5.75 ± 0.11	6.12 ± 0.12*

^*^P < 0.05,

^**^P < 0.01,

^***^P < 0.001 vs vehicle group. Data represents Mean ± SE, n = 5–6.

**Table 2:** Identification (LC-MS) of proteins in isolated Langerhan’s islets from rats exposed to vehicle or PCB126 (10 μg/kg).

SwissProt ID	Protein ID	Match ID	PCB/Vehicle ratio	Biological function
Response to oxidative stress				
Q66HD0	Heat shock protein 90 kDa (HSP90)	68	+5.94	Processing and transport of secreted proteins.
P13803	Electron transfer flavoprotein subunit alpha (Alpha-ETF)	16	+3.38	Electron acceptor for several dehydrogenases; transfer of electrons to mitochondrial respiratory chain.
P06761	78 kDa glucose-regulated protein (GRP78)	64	+1.60	Folding of proteins and degradation of misfolded proteins.
P19804	Nucleoside diphosphate kinase B (NDK B)	0	−2.48	Synthesis of nucleosides; cellular response to fatty acids, glucose, and oxidative stress.
Q05982	Nucleoside diphosphate kinase A (NDK A)	0	−2.48	Synthesis of nucleosides; cellular response to fatty acids, glucose, and drugs.
P29315	Ribonuclease inhibitor	42	−3.46	Inhibitor of RNAses; may play a role in redox homeostasis.
Carbohydrate metabolism				
Q9ER34	Aconitate hydratase	65	+4.47	Catalyses the isomerization of citrate to isocitrate.
P07943	Aldose reductase (AR)	22	+2.54	Reduction of carbonyl-containing compounds.
P00689	Pancreatic alpha-amylase	51	−1.62	Hydrolysis of alpha bonds of large alpha-linked polysaccharides.
P10760	Adenosylhomocysteinase	36	−2.25	Regulation of the intracellular concentration of adenosylhomocysteine.
Lipid metabolism				
P1 8886	Carnitine O-palmitoyltransferase 2 (CPT2)	61	+3.30	Carnitine O-palmitoyltransferase activity; fatty acid oxidation and transport.
P17764	Acetyl-CoA acetyltransferase	26	−5.26	Acetyl-CoA C-acetyltransferase activity; ketone body metabolism.
Cytoplasm/cytoskeleton proteins				
Q6IFW6	Keratin, type I cytoskeletal 10	2	+2.05	Belongs to the superfamily of structural proteins which form the intermediate filaments.
P60711	Actin, cytoplasmic 1	32	−1.46	Cell motility.
P63029	Translationally-controlled tumor protein (TCTP)	4	−2.47	Calcium binding and microtubule stabilization.
Digestive enzymes				
P08426	Cationic trypsin-3	5	−1.61	Serine-type endopeptidase activity.
P07338	Chymotrypsinogen B	12	−1.72	Serine-type endopeptidase activity.
P27657	Pancreatic triacylglycerol lipase	38	−2.11	Splits the esters of long-chain fatty acids to produce monoacylglycerol and free fatty acids.
P54316	Inactive pancreatic lipase-related protein 1	53	−2.64	Inhibitor of dietary triglyceride digestion.
Other proteins				
Q5U2Q3	Ester hydrolase C11orf54 homolog	19	+2.13	Exhibits ester hydrolase activity on the substrate p-nitrophenyl acetate.
P00731	Carboxypeptidase A1	33	−2.13	Catalyses the release of a C-terminal amino acid.
P10760	Adenosylhomocysteinase	36	−2.25	Regulation of the intracellular concentration of adenosylhomocysteine.

SwissProt ID refers to UniProt taxonomy Rattus norvegicus (http://www.uniprot.org/taxonomy/10116). Match ID refers to the pair number in 2D gel (Fig. 5a). Fold change in expression of proteins is presented as PCB126/vehicle ratio; (+) indicates up-regulated protein, (−) indicates down-regulated proteins in islets of Langerhans from rats exposed to PCB126.

## References

[b1] Nathan D. M. Diabetes: advances in diagnosis and treatment. JAMA 314, 1052–1062 (2015). 26348754 10.1001/jama.2015.9536

[b2] Lee D. H., Porta M., Jacobs D. R. Jr. & Vandenberg L. N. Chlorinated persistent organic pollutants, obesity, and type 2 diabetes. Endocr. Rev. 35, 557–601 (2014). 24483949 10.1210/er.2013-1084 PMC5393257

[b3] Thayer K. A., Heindel J. J., Bucher J. R. & Gallo M. A. Role of environmental chemicals in diabetes and obesity: a national toxicology program workshop review. Environ. Health Perspect. 120, 779–789 (2012). 22296744 10.1289/ehp.1104597 PMC3385443

[b4] Fisher B. E. Most unwanted. Environ. Health Perspect. 107, A18–A23 (1999). 9872725 10.1289/ehp.99107a18 PMC1566310

[b5] Mrema E. J. . Persistent organochlorinated pesticides and mechanisms of their toxicity. Toxicology 307, 74–88 (2013). 23219589 10.1016/j.tox.2012.11.015

[b6] Jepson P. D. . PCB pollution continues to impact populations of orcas and other dolphins in European waters Sci. Rep. 6, 18573 (2016). 26766430 10.1038/srep18573 PMC4725908

[b7] National Toxicology Program (NTP) Toxicology and carcinogenesis studies of 3,3′,4,4′, 5-pentachlorobiphenyl (PCB126) in female Harlan Sprague-Dawley rats (gavage studies). Nat. Toxicol. Program Tech. Rep. Ser. 520, 4–246 (2006). 16628245

[b8] Stockinger B., DiMeglio P., Gialitakis M. & Duarte J. H. The aryl hydrocarbon receptor: multitasking in the immune system. Annu. Rev. Immunol. 32, 403–432 (2014). 24655296 10.1146/annurev-immunol-032713-120245

[b9] Gregoris E. . Gas-particle distribution, sources and health effects of polycyclic aromatic hydorcarbons (PAHs), polychlorinated biphenyls (PCBs) and polychlorinated naphthalenes (PCNs) in Venice aerosols. Sci. Total Environ. 476-477, 393–405 (2014). 24486495 10.1016/j.scitotenv.2014.01.036

[b10] Herrick R. F., Stewart J. H. & Allen J. G. Review of PCBs in US schools: a brief history, an estimate of the number of impacted schools, and an approach for evaluating indoor air samples. Environ. Sci. Pollut. Res. Int. 10.1007/s11356-015-04574-8 (2015). PMC4635108 25940477

[b11] Lehmann G. M., Christensen K., Maddaloni M. & Phillips L. J. Evaluating health risks from inhaled polychlorinated biphenyls: research needs for addressing uncertainty. Environ. Health Perspect. 123, 109–13 (2015). 25302536 10.1289/ehp.1408564 PMC4314250

[b12] Rudel R. A. & Perovich L. J. Endocrine disrupting chemicals in indoor and outdoor air. Atmos. Environ. 43, 170–181 (2009). 10.1016/j.atmosenv.2008.09.025 PMC2677823 20047015

[b13] Shimada A. L. . Absorption of PCB126 by upper airways impairs G protein-coupled receptor-mediated immune response. Sci. Rep. 5, 14917 (2015). 26449762 10.1038/srep14917 PMC4598834

[b14] Gadupudi G., Gourronc F. A., Ludewig G., Robertson L. W. & Klingelhutz A. J. PCB126 inhibits adipogenesis of human preadipocytes. Toxicol. In Vitro 29, 132–141 (2015a). 25304490 10.1016/j.tiv.2014.09.015 PMC4250299

[b15] Gadupudi G. S. . PCB126-induced disruption in gluconeogenesis and fatty acid oxidation precedes fatty liver in male rats. Toxicol. Sci. 338, 1–7 (2015b). 10.1093/toxsci/kfv215 PMC4731404 26396156

[b16] Baker N. A. . Coplanar polychlorinated biphenyls impair glucose homeostasis in lean C57BL/6 mice and mitigate beneficial effects of weight loss on glucose homeostasis in obese mice. Environ. Health. Perspect. 121, 105–110 (2013). 23099484 10.1289/ehp.1205421 PMC3553436

[b17] Nyska A. . Exocrine pancreatic pathologic in female Harlan Sprague-Dawley rats after chronic treatment with 2,3,7,8-tetrachlorodibenzo-p-dioxin and dioxin-like compounds. Environ. Health Perspect. 112, 903–909 (2004). 15175180 10.1289/ehp.6869 PMC1242020

[b18] Lai I. K. . Does dietary copper supplementation enhance or diminish PCB126 toxicity in the rodent liver? Chem. Res. 26, 634–644 (2013). 10.1021/tx400049s PMC3660509 23527585

[b19] Rignall B. . Biological and tumor-promoting effects of dioxin-like and non-dioxin-like polychlorinated biphenyls in mouse liver after single or combined treatment. Toxicol. Sci. 133, 29–41 (2013). 23457121 10.1093/toxsci/kft034 PMC3627557

[b20] Suarez-Lopez J. R., Lee D. H., Porta M., Steffes M. W. & Jacobs D. R. Jr. Persistent organic pollutants in young adults and changes in glucose related metabolism over a 23-year follow-up. Environ. Res. 137, 485–494 (2015). 25706918 10.1016/j.envres.2014.11.001 PMC4429782

[b21] Ghosh S. . Biomarkers linking PCB exposure and obesity. Curr. Pharm. Biotechnol. 15, 1058–68 (2014). 25420728 10.2174/1389201015666141122203509 PMC4292903

[b22] Zong G., Grandjean P., Wu H. & Sun Q. Circulating persistent organic pollutants and body fat distribution: evidence from NHANES 1999–2004. Obesity 23, 1903–1910 (2015). 26237202 10.1002/oby.21161 PMC4551580

[b23] Agay-Shay K. . Exposure to endocrine-disrupting chemicals during pregnancy and weight at 7 years of age: a multi-pollutant approach. Environ. Health Perspect. 123, 1030–1037 (2015). 25956007 10.1289/ehp.1409049 PMC4590760

[b24] Santiago M. F., Pérez-Reyes P. L., López-Aparicio P., Recio M. N. & Pérez-Albarsanz M. A. Differential effects of PCBs on the induction of apoptosis machinery and PKCalpha translocation in rat renal tubular cell cultures. Toxicol. Lett. 163, 91–100 (2006). 16263226 10.1016/j.toxlet.2005.09.032

[b25] Lu P. . Activation of aryl hydrocarbon receptor dissociates fatty liver from insulin resistance by inducing fibroblast growth factor 21. Hepatology 61, 1908–1919 (2015). 25614121 10.1002/hep.27719 PMC4441569

[b26] Wang C. . Aryl hydrocarbon receptor deficiency enhances insulin sensitivity and reduces PPAR-α pathway activity in mice. Environ. Health Perspect. 119, 1739–1744 (2011). 21849270 10.1289/ehp.1103593 PMC3261983

[b27] Baker N. A. . Effects of adipocyte aryl hydrocarbon receptor deficiency on PCB-induced disruption of glucose homeostasis in lean and obese mice. Environ. Health Perspect. 123, 944–950 (2015). 25734695 10.1289/ehp.1408594 PMC4590748

[b28] Lee J. H. . A novel role for the dioxin receptor in fatty acid metabolism and hepatic steatosis. Gastroenterology 139, 653–663 (2010). 20303349 10.1053/j.gastro.2010.03.033 PMC2910786

[b29] Alexander D. L., Ganem L. G., Fernandez-Salguero P., Gonzalez F. & Jefcoate C. R. Aryl-hydrocarbon receptor is an inhibitory regulator of lipid synthesis and of commitment to adipogenesis. J. Cell. Sci. 111, 3311–3322 (1998). 9788873 10.1242/jcs.111.22.3311

[b30] Kerley-Hamilton J. S. . Obesity is mediated by differential aryl hydrocarbon receptor signaling in mice fed a Western diet. Environ. Health Perspect. 120, 1252–1259 (2012). 22609946 10.1289/ehp.1205003 PMC3440132

[b31] Wang M. L., Lin S. H., Hou Y. Y. & Chen Y. H. α-Naphthoflavone increases lipid accumulation in mature adipocytes and enhances adipocyte-stimulated endothelial tube formation. Nutrients 7, 3166–3183 (2015). 25942489 10.3390/nu7053166 PMC4446745

[b32] Arsenescu V., Arsenescu R. I., King V., Swanson H. & Cassis L. A. Polychlorinated biphenyl-77 induces adipocyte differentiation and proinflammatory adipokines and promotes obesity and atherosclerosis. Environ. Health Perspect. 116, 761–768 (2008). 18560532 10.1289/ehp.10554 PMC2430232

[b33] Kim M. J. . Inflammatory pathway genes belong to major targets of persistent organic pollutants in adipose cells. Environ. Health Perspect. 120, 508–514 (2012). 22262711 10.1289/ehp.1104282 PMC3339464

[b34] Myre M. & Imbeault P. Persistent organic pollutants meet adipose tissue hypoxia: does cross-talk contribute to inflammation during obesity? Obes. Rev. 15, 19–28 (2014). 23998203 10.1111/obr.12086

[b35] Cave M. . Polychlorinated biphenyls, lead, and mercury are associated with liver disease in American adults: NHANES 2003-2004. Environ. Health Perspect. 118, 1735–1742 (2010). 21126940 10.1289/ehp.1002720 PMC3002193

[b36] Mesnier A. . Transcriptional Effects of PCB118 and PCB153 on the liver, adipose tissue, muscle and colon of mice: highlighting of Glut4 and Lipin1 as main target genes for PCB induced metabolic disorders. PLoS One 10(6), e0128847 (2015). 26086818 10.1371/journal.pone.0128847 PMC4473719

[b37] Lee D. H. . Gamma-glutamyltransferase and diabetes–a 4 year follow-up study. Diabetologia 46, 359–364 (2003). 12687334 10.1007/s00125-003-1036-5

[b38] Ko S. H., Baeg. M. K., Han. K. D., Ko S. H. & Ahn Y. B. Increased liver markers are associated with higher risk of type 2 diabetes. World J Gastroenterol 21, 7478–7487 (2015). 26139993 10.3748/wjg.v21.i24.7478 PMC4481442

[b39] Wei D. . Association of serum gamma-glutamyl transferase and ferritin with the metabolic syndrome. J Diabetes Res. 2015, 741731 (2015). 26185768 10.1155/2015/741731 PMC4491402

[b40] Matthaei S., Stumvoll M., Kellerer M. & Häring H. U. Pathophysiology and pharmacological treatment of insulin resistance. Endocrin. Rev. 21, 585–618 (2000). 10.1210/edrv.21.6.0413 11133066

[b41] Kasuga M. Insulin resistance and pancreatic β cell failure. J. Clin. Invest. 116, 1756–1760 (2006). 16823472 10.1172/JCI29189 PMC1483164

[b42] Tsuura Y., Ishida H., Shinomura T., Nishimura M. & Seino Y. Endogenous nitric oxide inhibits glucose-induced insulin secretion by suppression of phosphofructokinase activity in pancreatic islets. Biochem. Biophysis. Res. Commun. 252, 34–38. (1998). 10.1006/bbrc.1998.9601 9813142

[b43] Bedoya F. J. . Regulation of pancreatic β-cell survival by nitric oxide: clinical relevance. Islets 4, 108–118 (2012). 22614339 10.4161/isl.19822

[b44] Kotas M. E. & Medzhitov R. Homeostasis, inflammation, and disease susceptibility. Cell 160, 816–827 (2015). 25723161 10.1016/j.cell.2015.02.010 PMC4369762

[b45] Spranger J. . Inflammatory cytokines and the risk to develop type 2 diabetes: results of the prospective population-based European Prospective Investigation into Cancer and Nutrition (EPIC)–Potsdam Study. Diabetes 52, 812–817 (2003). 12606524 10.2337/diabetes.52.3.812

[b46] Abdulla A., Awla D., Thorlacius H. & Regnér S. Role of neutrophils in the activation of trypsinogen in severe acute pancreatitis. J. Leukoc. Biol. 90, 975–982 (2011). 21810937 10.1189/jlb.0411195

[b47] Hartman H. . P-selectin mediates neutrophil rolling and recruitment in acute pancreatitis. Br. J. Surg. 99, 246–255 (2012). 22109627 10.1002/bjs.7775

[b48] Larance M. & Lamond A. I. Multidimensional proteomics for cell biology. Nat. Rev. Mol. Cell. Biol. 16, 269–80 (2015). 25857810 10.1038/nrm3970

[b49] Kavanagh K., Zhang L. & Wagner J. D. Tissue-specific regulation and expression of heat shock proteins in type 2 diabetic monkeys. Cell Stress Chaperones 14, 291–299 (2009). 18843550 10.1007/s12192-008-0084-7 PMC2728265

[b50] Yan F. . Role of HSP90 in biogenesis of the β-cell ATP-sensitive potassium channel complex. Mol. Biol. Cell 21, 1945–1954 (2010). 20427569 10.1091/mbc.E10-02-0116 PMC2883939

[b51] Uchiyama T. . Constitutively active heat shock factor 1 enhances glucose-driven insulin secretion. Metabolism 60, 789–798 (2011). 20817212 10.1016/j.metabol.2010.07.029

[b52] Lu H., Koshkin V., Allister E. M., Gyulkhandanyan A. V. & Wheeler M. B. Molecular and metabolic evidence for mitochondrial defects associated with β-cell dysfunction in a mouse model of type 2 diabetes. Diabetes 59, 448–459 (2010). 19903739 10.2337/db09-0129 PMC2809957

[b53] Sun X. . Di(2-ethylhexyl)phthalate-induced apoptosis in rat INS-1 cells is dependent on activation of endoplasmic reticulum stress and suppression of antioxidant protection. J. Cell Mol. Med. 20, 1–14 (2015). 10.1111/jcmm.12409 PMC4369815 25418486

[b54] Ahmed M., Forsberg J. & Bergsten P. Protein profiling of human pancreatic islets by two-dimensional gel electrophoresis and mass spectrometry. J. Proteome Res. 4, 931–940 (2005). 15952740 10.1021/pr050024a

[b55] Kowluru A. Defective protein histdine phosphorylation in islets from Goto-Karizaki diabetic rat. Am. J. Physiol. Endocrinol. Metab. 285, E498–E503 (2003). 12799314 10.1152/ajpendo.00121.2003

[b56] Veluthakal R., Suresh M. V. & Kowluru A. Down-regulation of expression and function of nucleoside diphosphate kinase in insulin-secreting beta-cells under *in vitro* conditions of glucolipotoxicity. Mol. Cell Biochem. 329, 121–129 (2009). 19367376 10.1007/s11010-009-0113-6

[b57] Monti D. M., Gesualdi N. M., Matousek J., Esposito F. & D’Alessio G. The cytosolic ribonuclease inhibitor contributes to intracellular homeostasis. FEBS Letters 581, 930–934 (2007). 17292889 10.1016/j.febslet.2007.01.072

[b58] Saha J. K., Xia J., Grondin J. M., Engle S. K. & Jakubowski J. A. Acute hyperglycemia induced by ketamine/xylazine anesthesia in rats: mechanisms and implications for preclinical models. Exp. Biol. Med. 230, 777–784 (2005). 10.1177/153537020523001012 16246906

[b59] Bonora E., Moghetti P., Zancanaro C., Cigolini M., Querena M., Cacciatori V., Corgnati A. & Muggeo M. Estimates of *in vivo* insulin action in man: comparison of insulin tolerance tests with euglycemic and hyperglycemic glucose clamp studies. J. Clin. Endocrinol. Metab. 68, 374–378 (1989). 2645308 10.1210/jcem-68-2-374

[b60] Monzillo L. U. & Hamdy O. Evaluation of insulin sensitivity in clinical practice and in research settings. Nutr Rev. 2003 61, 397–412 (2003). 10.1301/nr.2003.dec.397-412 14968910

[b61] Zhou X. & Stephens M. Efficient multivariate linear mixed model algorithms for genome-wide association studies. Nat. Methods 11, 407–409 (2014). 24531419 10.1038/nmeth.2848 PMC4211878

