# Supplementary material for: Long-term in vivo polychlorinated biphenyl 126 exposure induces oxidative stress and alters proteomic profile on islets of Langerhans
Source: Sci Rep. 2016 Jun 13;6:27882. doi: 10.1038/srep27882 (PMC4904407; doi:10.1038/srep27882)
Supplement: Supplementary Information [file srep27882-s1.pdf]

Long-term *in vivo* polychlorinated biphenyl 126 exposure induces oxidative stress and alters proteomic profile on islets of Langerhans

Rodrigo Azevedo Loiola[1]; Fabyana Maria dos Anjos[1]; Ana Lúcia Borges Shimada[1]; Wesley Soares Cruz[1]; Carine Cristiane Drewes [1]; Stephen Fernandes de Paula Rodrigues[1]; Karina Helena Morais Cardozo[2]; Valdemir Melechco Carvalho[2]; Ernani Pinto[1]; Sandra Helena Poliselli Farsky[1]*

[1]Department of Clinical and Toxicological Analyses, Faculty of Pharmaceutical Sciences, University of São Paulo, São Paulo, Brazil.

[2]Fleury Group, São Paulo, Brazil.

***Address correspondent author:** Sandra Helena Poliselli Farsky, Av. Prof. Lineu Prestes 580, Building 13B, Cidade Universitária. São Paulo, SP, Brazil. Postal Code 05508-900. Telephone: +55-11-3091-1193; Fax number: +55-11-3815-6593; e-mail: sfarsky@usp.br

1. Methods

1.1 Protocol of *in vivo* PCB180 exposure

During 15 days, once a day, the animals were anaesthetized with ketamine/xylazine solution (80:8 mg/kg respectively, intraperitoneally— i.p.) and exposed to PCB180 10 µg/Kg (Sigma-Aldrich, St. Louis, MO, USA) of body weight by intranasal instillation. Control animals received the equivalent volume of vehicle (saline solution containing 0.5% of dimethyl sulfoxide—DMSO). During exposure, body weight, food intake and water consumption were measured at every day.

1.2 Morphometry of the endocrine pancreas

Animals were perfused through the heart with fixation solution (PBS containing paraformaldehyde 4%—PFA 4%). The pancreas were excised and fixed (PFA 4%, 6 hr), followed by immersion in a cryoprotective solution (PBS containing sucrose 30%). Frozen pancreas sections (10 µm) were cut on a cryostat and stained with hematoxylin-eosin for morphometric analysis. Images were obtained by a microscopy Axiovert 100M (Carl Zeiss, Germany), and islets area (μm^2) was determined using the software AxioVision Release 4.8 (Carl Zeiss, Germany).

1.3 Cell viability and cell cycle

Islets were dissociated by incubation with trypsin 0.1% (5 min at 37ºC) before performing experiments. For evaluation of viability, cells were incubated with anti-annexin-V FITC-conjugated (1:100, 20 min, at 4ºC) and propidium iodate (PI, 50 µg/mL) immediately before analysis. For cell cycle, cells were incubated with 300 µL of fluorescent hypotonic solution [phosphate buffered solution (PBS), 2% fetal bovine serum (FBS), 0.05% Triton-X 100, 0.1% sodium citrate] containing RNAse A (15 mg/mL) and PI (50 mg/mL) for 30 min at room temperature. Data from 10.000 cell events were acquired in a FACSCanto II flow cytometer (BD, CA, USA) and analysed using software (FlowJo, BD, CA, USA).

2. Results

Figure S1

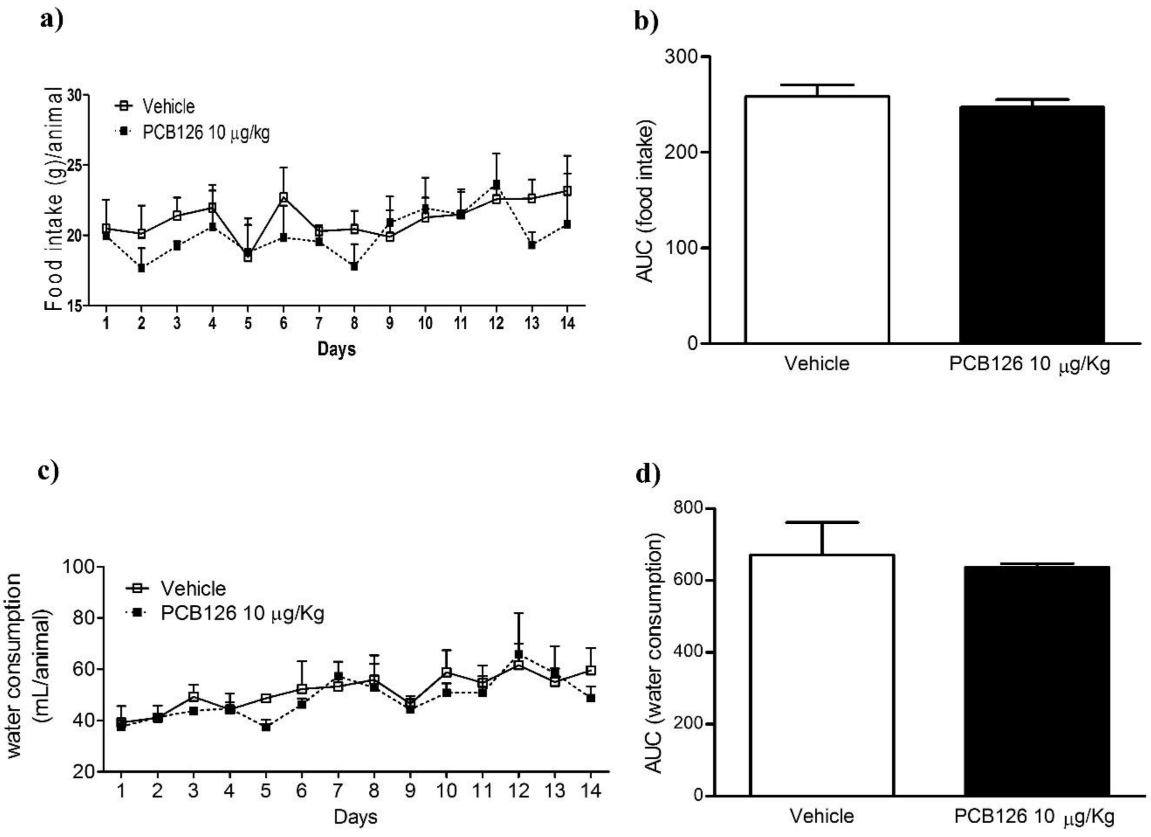

Figure S1: Effect of *in vivo* PCB126 exposure on food intake and water consumption. [A] Lines graph showing diary food intake (g/animal) and [B] bars graph showing AUC of total food intake (g) during PCB126 (10 µg/kg of body weight) or vehicle exposure. [C] Lines graph showing daily water consumption (mL/animal) and [B] bars graph showing AUC of total water consumption (mL) during PCB126 or vehicle exposure. Data were analyzed by two-way ANOVA [A and C] or one-way ANOVA [B and D].

Figure S2

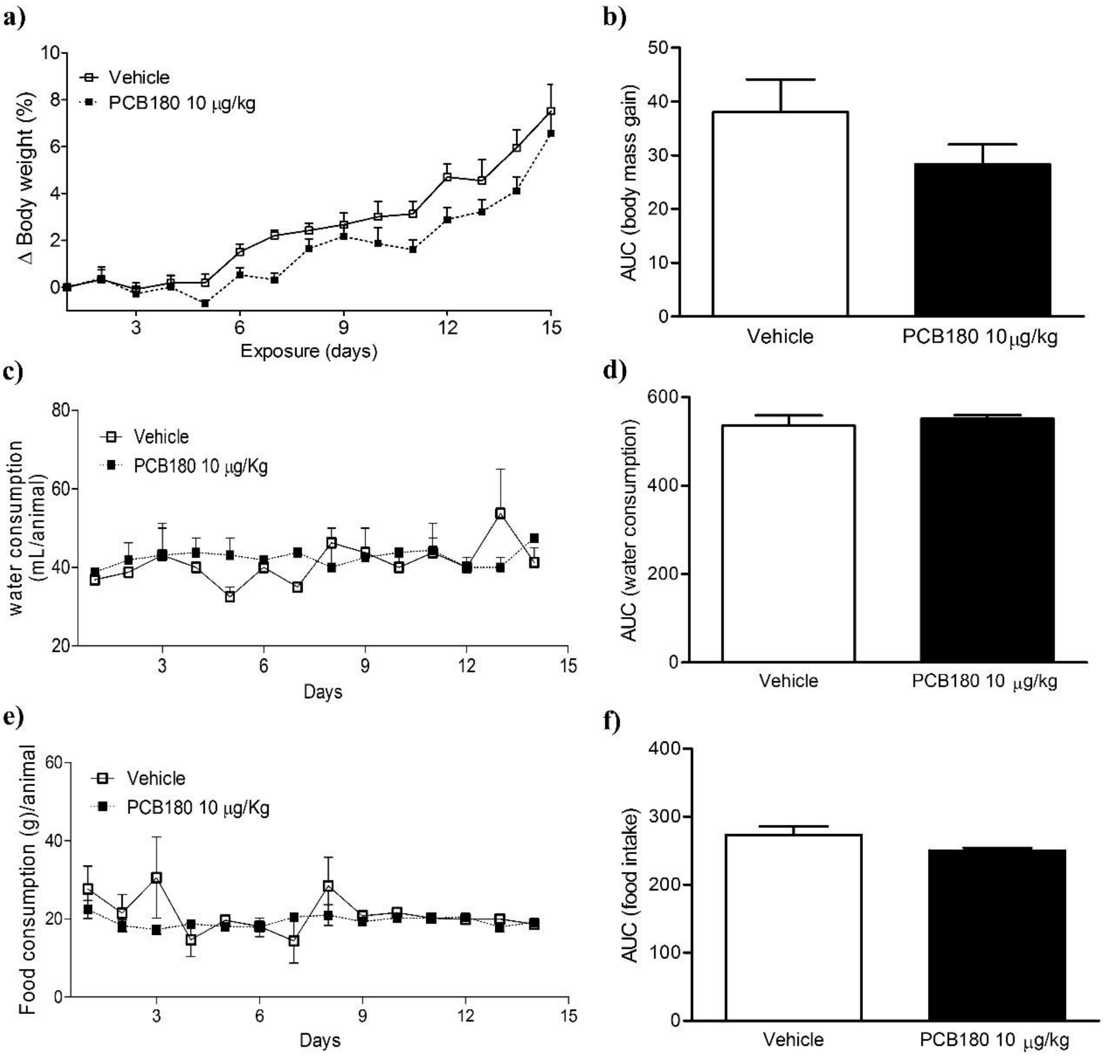

Figure S2: Effect of *in vivo* PCB180 exposure on weight gain, food intake, and water consumption. [A] Lines graph showing weight gain (% in relationship to the beginning of exposure) and [B] bars graph showing area under the curve (AUC) of body weight gain during PCB180 (10 µg/kg of BW) exposure. [C] Lines graph showing daily food intake (g/animal) and [D] bars graph showing area under the curve (AUC) of total food intake (g) during PCB180 (10 µg/kg of BW) or vehicle exposure. [E] Lines graph showing daily water consumption (mL/animal) and [F] bars graph showing area under the curve (AUC) of total water consumption (mL) during PCB180 or vehicle exposure. Data were analyzed by two-way ANOVA [A, C, and E] or student's *t*-test [B, D, and F].

Figure S3

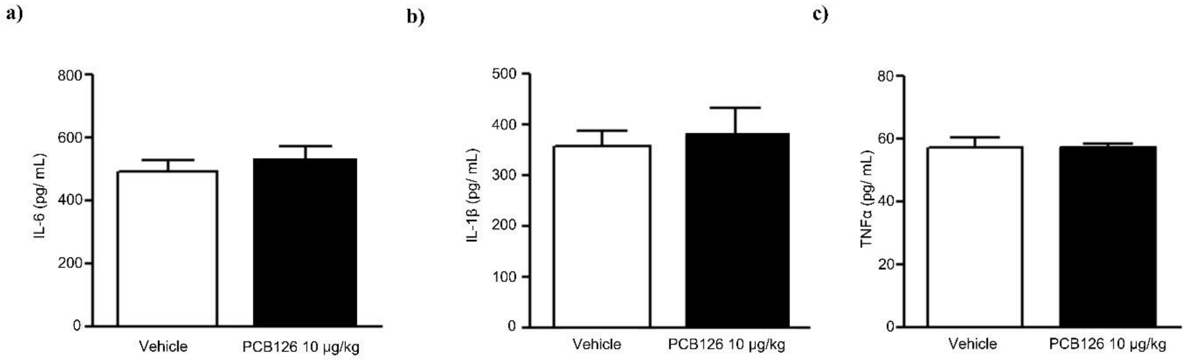

Figure S3: Effect of *in vivo* PCB126 exposure on plasmatic cytokines. Bars graph showing levels (pg/mL) of IL-1β [A], TNF-α [B], and IL-6 [C] on plasma from rats exposed to vehicle or PCB126 (10 µg/kg of body weight). Data were analyzed by student's *t*-test.

Figure S4

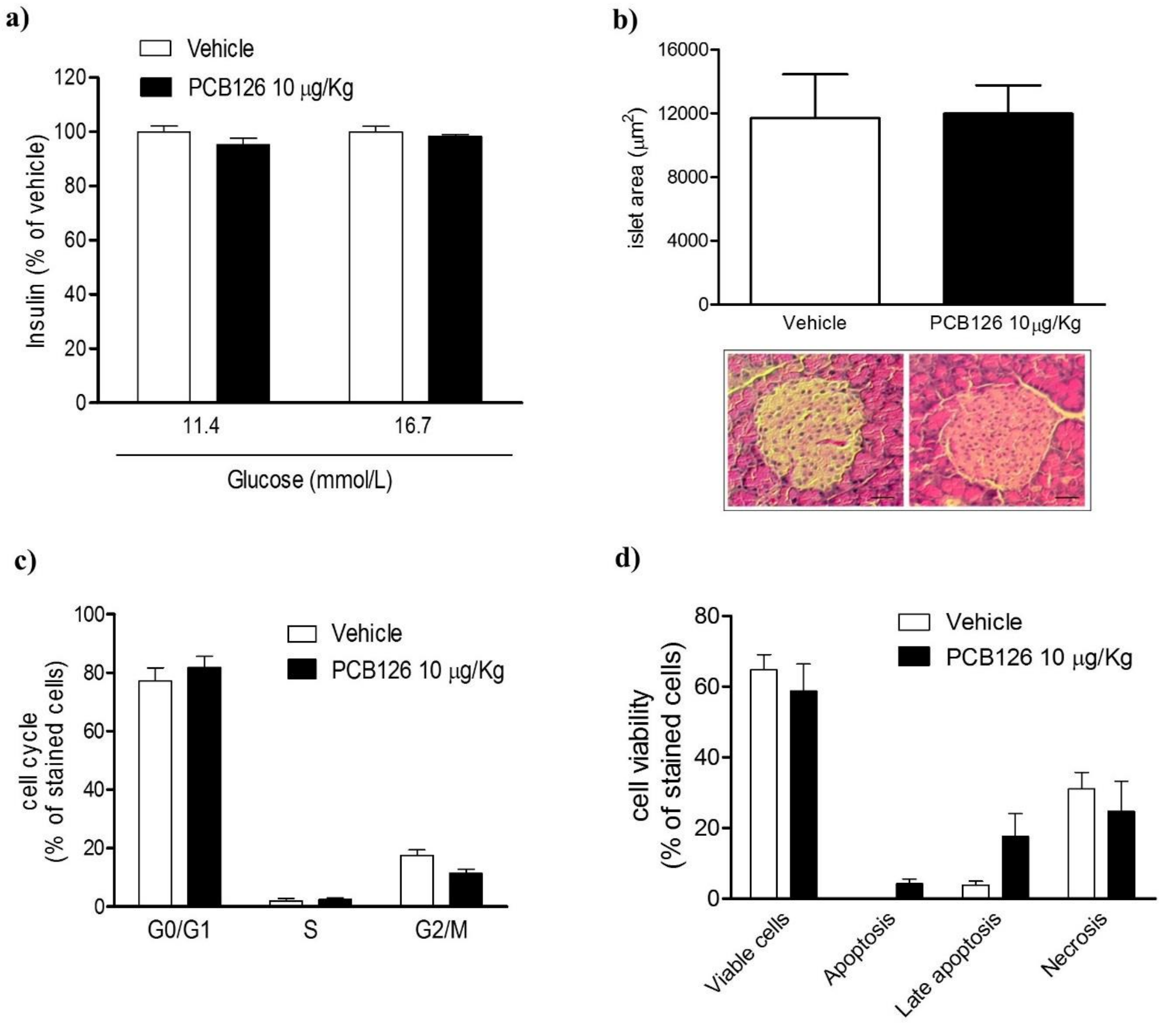

Figure S4: Effect of *in vivo* PCB126 exposure on function, morphology, and viability of islets of Langerhans. [A] Insulin secretion (% in relationship to vehicle group) by islets cultured at physiological (11.4 mM) or high glucose levels (16.7 mM). [B] Bars graph and representative image of islet morphology. Determination of cell cycle [C] and cell viability [D] of islets. Data were analyzed by two-way ANOVA [A, C-E] or student's *t*-test [B].
